# Evaluation of the Relationship between Baseline Autonomic Tone and Haemodynamic Effects of Dexmedetomidine

**DOI:** 10.3390/ph16030354

**Published:** 2023-02-25

**Authors:** Magdalena Wujtewicz, Paweł Twardowski, Tomasz Jasiński, Katarzyna Michalska-Małecka, Radosław Owczuk

**Affiliations:** 1 Department of Anaesthesiology and Intensive Therapy, Faculty of Medicine, Medical University of Gdańsk, 80-214 Gdańsk, Poland; 2 Department of Surgical Sciences, Dunedin School of Medicine, University of Otago, Dunedin 9016, New Zealand; 3 Department of Ophthalmology, Faculty of Medicine, Medical University of Gdańsk, 80-214 Gdańsk, Poland

**Keywords:** dexmedetomidine, heart rate variability, autonomic nervous system, hypotension, bradycardia

## Abstract

Dexmedetomidine, a central α-2 agonist, is used for procedural sedation and for conscious sedation influences on heart rate and blood pressure. Authors verified whether it is possible to predict bradycardia and hypotension with the use of heart rate variability (HRV) analysis for an autonomic nervous system (ANS) activity assessment. The study included adult patients of both sexes with an ASA score of I or II scheduled for ophthalmic surgery to be performed under sedation. The loading dose of dexmedetomidine was followed by a 15 min infusion of the maintenance dose. The frequency domain heart rate variability parameters from the 5-min Holter electrocardiogram recordings before dexmedetomidine administration were used for the analysis. The statistical analysis also included pre-drug heart rate and blood pressure as well as patient age and sex. The data from 62 patients were analysed. There was no relationship between the decrease in heart rate (42% of cases) and initial HRV parameters, haemodynamic parameters or sex and age of patients. In multivariate analysis, the only risk factor for a decrease in mean arterial pressure (MAP) > 15% from the pre-drug value (39% of cases) was the systolic blood pressure before dexmedetomidine administration as well as for a >15% decrease in MAP sustained at more than one consecutive time point (27% of cases). The initial condition of the ANS did not correlate with the incidence of bradycardia or hypotension; HRV analysis was not helpful in predicting the abovementioned side effects of dexmedetomidine.

## 1. Introduction

Dexmedetomidine is a well-known drug that is used for conscious sedation in intensive care unit (ICU) patients and for procedural sedation during surgical or diagnostic procedures [1]. Initially, dexmedetomidine was approved by the FDA for sedation in ICU patients in 1999; nine years later, it was approved for procedural sedation [2]. Off-label uses include the treatment and prevention of delirium, adjunctive analgesia, as therapy for insomnia in the ICU, and as treatment for alcohol withdrawal. Dexmedetomidine has also been used in peripheral nerve blocks to prolong the duration of analgesia [3,4,5], as it acts through central α-2 mimesis, causing sedation, analgesia, and anxiolysis. The administered drug produces rapid and stable sedation, while maintaining a high degree of patient reusability and anxiety reduction. Typical side effects are a decrease in arterial pressure and heart rate [6]. The abovementioned central hypotensive and bradycardic mechanism results from the postsynaptic activation of α_2_ adrenoceptors in the central nervous system, which inhibits sympathetic activity [7,8]. Due to that feature, it can attenuate stress responses to surgical manipulations [9]. However, in some patients, its sympatholytic action may be undesirable. Those who are initially hypotensive and/or have a slow heart rate may become more hypotensive and/or present with severe bradycardia upon administration, and this side-effect concerns both surgical and ICU patients. Since dexmedetomidine influences heart rate variability (HRV), as shown by Tarvainen et al. [10], it is possible that the tension of the sympathetic and/or parasympathetic component of the autonomic nervous system (ANS) before drug administration may affect the circulatory effect it exerts.

The aim of the study was to determine whether the baseline status of the ANS determines the haemodynamic effect of dexmedetomidine.

## 2. Results

**Figure 1:**
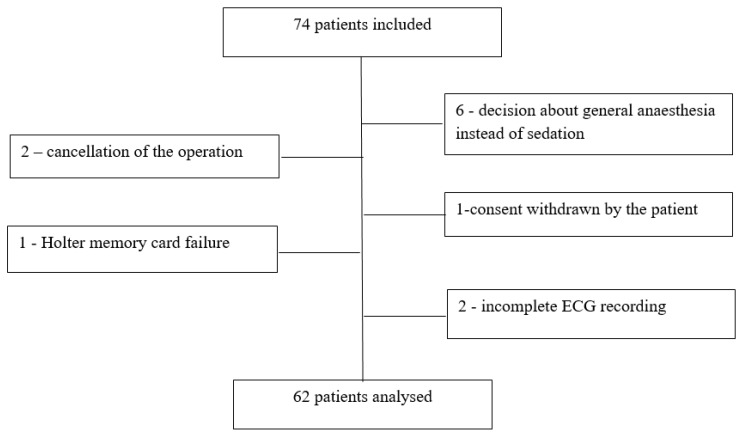
Patients’ flowchart.

Although 74 patients were included in this study, there were 12 dropouts (Figure 1).

The reasons for the dropouts were as follows: (1) general anaesthesia used instead of sedation because of surgical reasons, (2) withdrawal of consent by the patient, (3) cancellation of the operation, (4) incomplete ECG recording, and (5) no repairable damage to the Holter memory card.

Ultimately, the data of 62 patients were analysed and characteristics of the study participants are presented in Table 1.

HRV parameters that were analysed were low frequency (LF) and high frequency (HF) changes and the LF/HF ratio. Haemodynamic parameters that were analysed were heart rate (HR) and systolic arterial pressure (SAP), diastolic arterial pressure (DAP), and mean arterial pressure (MAP). The values of haemodynamic and HRV parameters before dexmedetomidine administration are shown in Table 2.

Haemodynamic values at six time points, including before dexmedetomidine infusion and during loading and maintenance infusions, are presented in Table 3.

The HR decreased by more than 20% from the pre-drug values in 26 cases (42%). There was no relationship between this phenomenon and initial HRV parameters, haemodynamic parameters or sex, and age of patients. Table 4.

A decrease in MAP of more than 15% from the pre-drug value and a decrease in MAP of more than 15% from the pre-drug value, sustained for more than one consecutive time point, were observed in 24 (39%) and 17 cases (27%), respectively (Table 5).

In the multivariate analysis, the only risk factor for a decrease in MAP > 15% was SAP before dexmedetomidine administration (SAP LI 0) (OR = 1.060; 95% CI: 1.011–1.113; *p* = 0.016).

In the multivariate analysis, the only risk factor for a decrease in MAP > 15% sustained at more than one consecutive time point was SAP LI 0. (OR = 1.056; 95% CI: 1.005–1.109; *p* = 0.028).

## 3. Discussion

Dexmedetomidine, a selective α-2 receptor agonist indicated for procedural sedation and sedation of ICU patients, has a wide range of pharmacological properties. It exerts sympatholytic effects by inhibiting the release of norepinephrine in sympathetic nerve endings [11,12]. Vasoconstriction and sympatholysis lead to typical haemodynamic effects, such as bradycardia and transient hypertension followed by hypotension. Sedative actions are achieved via the activation of α-2 receptors in the locus coeruleus [3].

Minimally invasive surgical procedures that do not cause intensive pain or when pain can be managed with topical medications are often performed under conscious sedation. This allows us to preserve cooperation between the operator and the patient. Analgesia is a basis for such cooperation and dexmedetomidine exerts an analgesic effect, either given systematically or as an adjuvant to regional anaesthesia, as well as an opioid-sparing effect [13,14,15,16,17].

Effects on the cardiovascular system depend on the dose administered. At lower infusion rates, systemic effects predominate, leading to a decrease in HR and blood pressure; this is a commonly known side effect linked to the drug. At higher doses, peripheral vasoconstrictive effects predominate, leading to an increase in vascular resistance in the systemic circulation and blood pressure, with the effect of a slowing HR still intensifying [18]. Additionally, as a result, a typical side effect of dexmedetomidine administration is a decrease in HR. Its bradycardic effect may result not only from its central α-2 blockade but also from inhibition of the acetylcholinesterase receptor channels and sodium channels. This potential central antisympathetic activity of dexmedetomidine was observed in rats by Yang et al. [19].

Preoperative HRV may be a useful tool in the prediction of some perioperative outcomes in general and spinal anaesthesia, as described by Frandsen et al. [20,21]. A pioneering method of monitoring the depth of anaesthesia using, among other things, physiological parameters and HRV, has been recently described by Kenwirth et al. [22] a few years ago, showing the potential use of HRV assessment in the perioperative period. As shown by Fujiwara et al. [23], HRV, namely the LF/HF ratios, were significantly correlated with HR increase caused by tracheal intubation. In the present study, the association between HRV recorded before dexmedetomidine administration and the incidence of bradycardia was checked. No correlation was found in the previous study.

The incidence of bradycardia in the present study was 42%, and the HR recorded was statistically lower than the pre-drug value at all time points.

In a study by Inagaki et al. [24], the incidence of bradycardia was 26.4% and 30.4%, depending on the loading dose of 0.5 µg kg^−1^ and 1 µg kg^−1^, respectively. The criterion used was a 30% decrease in HR or <40 beats min^−1^. In another study that included elderly patients, bradycardia occurred in 43% of patients. Bradycardia was defined as a HR below 50 beats min^−1^ [25]. We decided not to use a single cut-off value for bradycardia, but a decrease by 20% from the pre-drug value. Since some patients may have initially low heart pressure, a decrease from a value is clinically important.

According to the abovementioned study, it must be highlighted that the comparison of the incidence of bradycardia among different studies is difficult because of varying criteria used to define bradycardia.

What is clinically important is that bradycardia or an excessive decrease in the HR may have undesirable clinical sequelae. It is worth to mention that cardiac arrest can occur when a patient receives additional drugs that decrease HR and attenuate sympathetic outflow, for instance, pregabalin; Aikaterini et al. [26] reported such a complication in a patient operated on for cataract treatment, a relatively safe procedure, performed under dexmedetomidine sedation. During the present study, we did not observe any unfavourable consequences of the decrease in HR.

Another feature of dexmedetomidine is its influence on ANS activity.

Hogue et al. [27] found reduced sympathetic activity with dexmedetomidine infusions during the control period compared with placebo treatment to elicit little change in cardiac parasympathetic modulation. A similar conclusion was made by Cho et al. [28] in their study, in which dexmedetomidine attenuated pneumoperitoneum and surgical stress-induced sympathetic activation. This feature may be particularly useful in patients at risk for cardiac morbidities in whom adverse responses to surgical stressors can be disadvantageous.

Bradycardia can be problematic not only in surgical patients, but also in ICU patients, and dexmedetomidine is commonly used as a sedative in ICU patients. In the ICU, patients often do not require deep sedation. Except in specific situations, such as severe head trauma and severe acute respiratory distress syndrome, the patient should be calm, rousable, and able to communicate. Moreover, according to the Guidelines for the Prevention and Management of Pain, Agitation/Sedation, Delirium, Immobility, and Sleep Disruption in Adult Patients in the ICU, light vs. deep sedations are suggested. Dexmedetomidine, with its anxiolytic, sedative, and analgesic effects, has become a sedative drug of choice (equally to propofol) over benzodiazepines for sedation in critically ill, mechanically ventilated adults. For delirium treatment in mechanically ventilated adults where agitation precludes weaning from mechanical ventilation or extubation, dexmedetomidine use has been suggested [29].

However, despite its desirable effects on ICU population, the dexmedetomidine sympatholytic effect can even lead to cardiac arrest in ICU patients without cardiovascular comorbidities, just after vagal stimulation, as described in a report by Bahraini et al. [30].

With respect to the above, we searched for the potential relationship between basic autonomic tone and the incidence of bradycardia, and no relationship was found in the present study. Additionally, there was also no relationship between bradycardia and initial HR or arterial blood pressure.

According to the literature, HRV is a sensitive method for detecting individuals who may be at risk of BP instability during general anaesthesia [31]. Similar findings were published 12 years later by Padley et al. [32], who concluded that HRV was a useful screening tool in identifying patients undergoing major abdominal surgery who were at risk of haemodynamic instability after anaesthesia induction.

With this knowledge, we assessed whether other components of HRV before dexmedetomidine administration can help to predict the occurrence of hypotension caused by dexmedetomidine, and no correlation was found. On the other hand, in the abovementioned Padley study, hypotension was defined as a more severe decrease in SAP or MAP—more than a 30% drop from baseline or a MAP ≤ 60 mm Hg.

As mentioned above, the influence of dexmedetomidine on arterial blood pressure has two features. A bolus dose, when the plasma concentration is high, causes an increase in blood pressure (and a concomitant, baroreceptor-mediated, decrease in heart rate), which results from an increase in systemic vascular resistance (vascular α-2 receptor activation). Hypertension may be severe, with systolic blood pressure exceeding 200 mm Hg [33].

As the plasma concentration decreases, a hypotensive phase occurs. This is caused by vasodilation resulting from activation of the α_2_-receptors in the vascular endothelial cells acting together with presynaptic α_2_-adrenoreceptors, inhibiting the sympathetic release of catecholamines [11,12,34].

The incidence of hypotension in our study was 39%, and hypotension lasting for more than 5 min was found in 27% of cases. We did not observe hypertension during the loading dose.

This effect, caused by the activation of the peripheral postjunctional α-1 adrenergic receptors, is seen when intravenous infusion of high doses or when rapid intravenous bolus is administered. This occurs because dexmedetomidine loses its α-2 receptor selectivity when given in a high dose or as a rapid bolus injection. Additionally, transient activation of peripheral α-2B is responsible for hypertensive effects [18].

The loading dose was given within 10 min, according to the product characteristic—this can be the reason for not observing hypertension. Another reason could be the use of non-invasive blood pressure measurement and the measures in 5 min periods. Sudden, transient blood pressure changes are easier to detect when using continuous measurement.

In our study, blood pressure (SAP, DAP, MAP) was significantly lower than the pre-administration values at 10 min of loading dose infusion and during 15 min of the maintenance dose. It was found that the only risk factor for hypotension was systolic blood pressure before dexmedetomidine administration.

The hypotensive effect may be desirable in some situations; for instance, dexmedetomidine has been used intraoperatively to facilitate controlled hypotension [35,36]. Even if dexmedetomidine is more effective than magnesium sulfate, it must be kept in mind that the risk of bradycardia is higher [37].

The present study included relatively healthy patients scheduled for a planned procedure. This population differs from critically ill patients, in whom dexmedetomidine is used commonly.

The clinical characteristics make dexmedetomidine a useful drug in indications other than just sedation; however, these uses, including treatment and prevention of delirium, adjunctive analgesia, therapy for insomnia in the ICU, and treatment of alcohol withdrawal, are off-label [29,38,39,40]. ICU patients are often hypotensive, sometimes bradycardic, and often have comorbidities and receive several drugs that influence haemodynamics and/or the ANS.

However, dexmedetomidine is used for sedation in this population. The incidence of dexmedetomidine-induced hypotension is similar to that observed in surgical patients.

In a study of critically ill patients by Gerlach et al. [41], hypotension occurred with an incidence of 42.8%. A history of coronary artery disease and higher acuity were identified as independent risk factors for dexmedetomidine-associated hypotension.

In our study, the only independent risk factor for hypotension development was systolic blood pressure before dexmedetomidine administration. However, the population studied is totally different from the ICU population.

In patients in the cardiovascular ICU, hypotension was also observed in 42% of patients. Bradycardia was found to be an independent predictor of hypotension [42].

It is worth mentioning that on multivariable adjusted analysis, it was found that dexmedetomidine sedation was associated with lower vasopressor requirements to maintain the target MAP in septic shock patients in the first 48 h. The potential bias was deeper sedation in the usual care group, but according to the authors, the findings are exploratory [43].

In a systematic review, it was shown that dexmedetomidine was effective in reducing both the ICU length of stay and time to extubation. The use of dexmedetomidine, however, was associated with an increased risk of bradycardia, but not with overall mortality [44].

Despite haemodynamic side effects, taking into account that dexmedetomidine is safe in terms of the risk of respiratory depression, Weerink et al. [3], in their literature review, concluded that dexmedetomidine is an efficacious and safe drug for the sedation of ICU patients and/or for use during procedural sedation [3]. As such, this drug is used in almost all procedural and intensive care settings [45].

On the other hand, in new data recently published this year, dexmedetomidine was associated with an increased risk of mortality in the age group ≤ 65 years compared with alternative sedatives (odds ratio 1.26; 95% credibility interval 1.02 to 1.56). The reason for this is not yet known [46].

In the anaesthesia field, monitoring plays an essential role. Changes in the activity of the ANS measured as heart rate variability changes caused by drugs used for anaesthesia might one day become a part of some algorithms. A pioneering method of monitoring the depth of anaesthesia, using, among other things, physiological parameters and HRV, has been described by Kenwirth et al. [22]. It can be assumed that in the future, anaesthesia depth monitors will become more and more sophisticated. It cannot be ruled out that cardiorespiratory interactions and ANS activity will play pivotal roles. In this perspective, additionally to the “pure” influence of sedative drugs on cardiac parameters, the use of the HRV method for the “total effects” of drugs used in anaesthesia may be very helpful, and from this perspective the method used for this study adds additional value.

The study has some limitations. Since it includes relatively healthy patients, without comorbidities and who are not taking drugs that influence the ANS, the results cannot be extrapolated to more sick patients. At the same time, creating a study with a relatively homogenous population with comorbidities could be extremely difficult and at the same time not feasible for analysis purposes.

The next limitation is the sample size (n = 62 analysed patients). The assumptions that MAP decreases > 15% and HR decreases > 20% and will be clinically significant were made.

Another limitation is the use of noninvasive blood pressure monitoring. Direct measurement would be more precise for the detection of sudden, transient blood pressure changes. However, this kind of monitoring is the standard method of intraoperative blood pressure monitoring in patients with an ASA score of I–II and during uncomplicated operations [47].

Bias may also be caused by the fact that during maintenance dose infusion, there was no strictly defined dose volume prescribed for all the patients—the infusion rate was given to maintain a Ramsay score of between 2–3.

In conclusion, in the analysed setting, the incidence of bradycardia was relatively high and similar to that reported in the literature. There was no relationship found between the incidence of bradycardia and the analysed haemodynamic and the HRV parameters and demographic characteristics of patients. Hypertension during the loading dose was not observed. Hypotension was common and was recorded at 10 min of loading dose infusion and lasted until the end of the study. The only independent risk factor for hypotension development was systolic blood pressure before dexmedetomidine administration. The initial condition of the ANS did not correlate with the incidence of bradycardia or hypotension; HRV analysis was not helpful in predicting the abovementioned side effects of dexmedetomidine.

## 4. Materials and Methods

Ethical approval for this study was provided by the Independent Committee for Scientific Research at the Medical University of Gdansk, Gdansk, Poland (NKEBN/266/2014).

This study was registered at ClinicalTrials.gov (Identifier NCT02566863). Written informed consent was obtained from all patients.

The study included adult patients of both sexes with an ASA score of I or II scheduled for ophthalmic surgery to be performed under sedation.

The exclusion criteria were: a lack of consent to participate in the study, contraindications to the use of dexmedetomidine, history of hypersensitivity to dexmedetomidine, advanced heart block (second or third degree), if cardiac pacing is not used, uncontrolled hypotension, acute cerebrovascular disease, the use of drugs with known effects on the ANS, and coexistence of diseases that modulate autonomic system activity.

Data analysed were an incidence of a decrease in heart rate (HR) of more than 20% from the values before dexmedetomidine administration, a decrease in mean arterial pressure (MAP) of more than 15% from the pre-drug value, and a MAP decrease of more than 15% from the pre-drug value that persisted for more than one consecutive time point.

The definition of perioperative hypotension differs between studies and varies from a 10 to 60% drop from the baseline values [48].

The relationship of occurrence was assessed for the abovementioned outcomes in relation to heart rate variability, haemodynamic parameters recorded before dexmedetomidine administration, and demographic parameters.

In the ophthalmology ward, an ECG Holter monitor with a digital sampling rate of 180/s/s/channel (H3+, Mortara Rangoni Europe, Bologna, Italy) was connected to the patient approximately 30 min before the planned initiation of anaesthesia, and recording commenced. Once the patient was transferred to the operating suite, standard monitoring of vital functions was initiated (HR, non-invasive measurement of arterial pressure, arterial blood saturation, respiration rate). Patients were given oxygen via a face mask and Holter readings were recorded automatically.

A loading dose (LI) of 1 µg kg^−1^ body weight of dexmedetomidine (Dexdor, Orion Pharma, Finland) was given within 10 min, followed by titrated continuous infusion (range 0.2 to 1 µg kg^−1^ h^−1^) with the aim of a Ramsay sedation score of between 2–3. Haemodynamic parameters were noted before dexmedetomidine administration, after 5 and 10 min of loading dose infusion, and after 5, 10, and 15 min of maintenance dose infusion.

For the assessment of autonomic tone activity, 5-min recordings before the administration of dexmedetomidine were taken into analysis.

During this period of 25 min of drug infusion (maintenance dose, MI), no surgical procedures were performed. After the drug infusion, the study was stopped, and the surgical procedure began.

Heart rate variability (HRV) in the frequency domain was analysed using a nonparametric model (fast Fourier transformation) over 256 RR intervals. ECG recordings were automatically imported into HRV ver. 1.1 for analysis using H-Scribe (Mortara Rangoni Europe, Bologna, Italy).

There is no consensus on how to analyze HRV in the frequency domain. Two alternative approaches are nonparametric fast Fourier transformation (FFT) or parametric autoregressive model (AR) [49]. Both methods have their perks. We decided to use FFT because it is more straightforward and less computationally challenging. However, it must be noted that the AR model has a more accurate estimate of the power spectral density [50]. Studies also suggest that there is often a correlation between obtaining results from the FFT and AR models. HRV analysis with the FFT model is a commonly accepted procedure in HRV assessment [51,52,53].

Parasympathetic pulsation is thought to be reflected by high frequency (HF) values, while the interpretation of low-frequency (LF) changes is not as clear. Despite the fact that both sympathetic and parasympathetic components are represented by LF changes, the LF component has been deemed responsible for sympathetic changes. As a result of the above argumentation, the LF/HF ratio reflects the balance between sympathetic and parasympathetic activity [54,55]. An LF/HF ratio > 2 indicates sympathetic activation and a reduced vagal tone [56].

Statistical analysis was performed using Prism 9 software (GraphPad, Boston, MA, USA). The normality of the continuous data distribution was tested with the Shapiro–Wilk test. Continuous variables with a normal probability distribution are presented as the mean with a 95% confidence interval (CI). For the continuous variables with a different probability distribution, the median, the interquartile range (IQR), and the range are given. Risk factors for bradycardia and hypotension were determined using the calculation odds ratio (OR) and 95% CI for the independent predictors using multiple logistic regression. Regression analysis included parameters with *p* < 0.20 in the univariate analysis. *p* < 0.05 was considered statistically significant.

## 5. Conclusions

The initial condition of the ANS did not correlate with the incidence of bradycardia or hypotension; HRV analysis was not helpful in predicting the abovementioned side effects of dexmedetomidine.

## Figures and Tables

**Table 1:** Characteristics of study patients (n = 62). Values are number or mean (95% CIs) or median (IQR [range]).

Female sex	31
Age; years	54 (42–60 [25–65])
Height; cm	171.4 (95% CI: 168.8–174.0)
Body mass; kg	77.8 (95% CI: 73.5–82.2)
ASA physical status 1	50

**Table 2:** Haemodynamic and heart rate variability parameters before DEX administration. Values are mean (95% CIs) or median (IQR [range]).

Systolic Arterial Pressure; mmHg	137 (95% CI: 133–141)
Diastolic arterial pressure; mmHg	81 (95% CI: 79–84)
Mean arterial pressure; mmHg	100 (95% CI: 97–102)
Heart rate; beats min^−1^	69 (95% CI: 66–72)
Low frequency; ms^2^	1206 (324–1617 [56–5093])
High frequency; ms^2^	421 (89–578 [8–2626])
Low frequency/high frequency ratio	4.59 (2.19–5.91 [0.11–19.65])

**Table 3:** Haemodynamic values at 6 time points- before dexmedetomidine infusion and during loading and maintenance infusions. Values are mean (95% CI).

	LI 0	LI 5	LI 10	MI 5	MI 10	MI 15
Heart rate; beats min^−1^	69 (67–72)	63 * (60–67)	59 * (56–62)	60 * (58–63)	60 * (58–63)	59 * (57–62)
SAP; mmHg	137 (133–141)	133 (129–136)	131 ** (127–135)	128 *** (124–132)	124 *** (120–128)	123 *** (119–127)
DAP; mmHg	81 (79–84)	79 (76–81)	77 † (74–80)	76 †† (73–78)	74 †† (71–76)	74 †† (71–77)
MAP; mmHg	100 (91–102)	97 (94–99)	95 ‡ (92–98)	90 ‡‡ (85–95)	91 ‡‡ (88–94)	90 ‡‡ (87–93)

SAP—systolic arterial pressure, DAP—diastolic arterial pressure, MAP—mean arterial pressure. LI—loading infusion; MI—maintenance infusion; LI 0—before dexmedetomidine administration; LI 5, LI 10—at 5th and 10th minute of loading dose infusion, respectively; MI 5, MI 10, MI 15—at 5th, 10th and 15th minute of maintenance dose infusion. * *p* < 0.00005 compared to heart rate at LI 0; ** *p* = 0.005 compared to SAP LI 0; *** *p* < 0.00005 compared to SAP LI 0; † *p* < 0.005 compared to DAP LI 0; †† *p* < 0.00005 compared to DAP LI 0; ‡ *p* = 0.0006 compared to MAP LI 0; ‡‡ *p* < 0.00005 compared to MAP LI 0.

**Table 4:** Decrease in heart rate for more than 20% from the values before dexmedetomidine administration (n = 26) in relation to analysed parameters.

Variable	Odds Ratio (95% CI)	*p* Value
Low frequency	0.999 (0.998–1.000)	0.2233
High frequency	1.001 (0.999–1.004)	0.2215
Low frequency/high frequency ratio	1.050 (0.887–1.266)	0.5720
Heart rate	1.064 (1.005–1.134)	0.0385 *
Mean arterial pressure	0.942 (0.877–1.005)	0.0788
Male sex	0.930 (0.211–4.043)	0.9216
Age	0.981 (0.926–1.039)	0.5148

* *p* < 0.05.

**Table 5:** The decrease in MAP for more than 15% from the pre-drug value (n = 24) and MAP decrease for more than 15% from the pre-drug value, sustained for more than one consecutive time point (n = 17) in relation to analysed parameters. *p*-values are shown.

Variable	Decrease MAP >15% from the Pre-Drug Value		MAP Decrease >15% from the Pre-Drug Value, Observed at More Than One Consecutive Time Point	
	Odds Ratio (95% CI)	*p* Value	Odds Ratio (95% CI)	*p* Value
Low frequency	1.000 (0.999–1.001)	0.6691	1.000 (0.999–1.000)	0.1504
High frequency	0.998 (0.995–0.999)	0.0245 *	0.997 (0.994–0.999)	0.0388 *
Low frequency/high frequency ratio	1.186 (1.022–1.429)	0.0433 *	0.920 (0.682–0.999)	0.4932
Heart rate	0.995 (0.930–1.060)	0.8714	1.018 (0.970–1.069)	0.468
Systolic arterial pressure	1.065 (1.024–1.115)	0.0033 *	1.057 (1.015–1.106)	0.0107 *
Diastolic arterial pressure	1.046 (0.991–1.111)	0.1173	1.017 (0.961–1.080)	0.5528
Mean arterial pressure	1.079 (1.020–1.154)	0.0140 *	1.052 (0.994–1.122)	0.0926
Male sex	0.9295 (0.2105–4.043)	0.9216	0.924 (0.845–0.998)	0.974
Age	0.9814 (0.926–1.039)	0.5149	0.993 (0.944–1.046)	0.7956

* *p* < 0.05.

## Data Availability

Not applicable.
